# Can our experience with surveillance for inherited pancreatic cancer help to identify early pancreatic cancer in the general population?

**DOI:** 10.1007/s10689-024-00363-6

**Published:** 2024-03-05

**Authors:** J.-Matthias Löhr, Daniel Öhlund, Emma Söreskog, Emil Andersson, Miroslav Vujasinovic, Niklas Zethraeus, Malin Sund

**Affiliations:** 1 Karolinska Comprehensive Cancer Center and Karolinska Institutet, Stockholm, Sweden; 2 Department of Radiation Sciences and Wallenberg Centre for Molecular Medicine (WCMM), Umeå University, Umeå, Sweden; 3 Department of Learning, Informatics, Management and Ethics (LIME), Karolinska Institutet, Stockholm, Sweden; 4 Department of Surgery, University of Helsinki and Helsinki University Hospital, Helsinki, Finland; 5 Department of Surgical and Perioperative Sciences/ Surgery, Umeå University, Umeå, Sweden; 6 Div. of Surgery & Oncology, Dept. of Upper Abdominal Diseases, CLINTEC Karolinska Institutet, Karolinska Comprehensive Cancer Center, Stockholm, SE-141 86 Sweden

**Keywords:** Pancreatic cancer, Surveillance, General population, Screening, Individuals at risk, Incidental finding

## Abstract

Screening of the general population for cancer is a matter of primary prevention reducing the burden of disease. Whilst this is successful for several cancers including breast, colon and prostate, the situation to screen and hence prevent pancreatic cancer is different. The organ is not as accessible to simple physical exam or biological samples (fecal or blood test). Neither exists a blood test such as PSA that is cost-effective. Reviewing the evidence from screening risk groups for pancreatic cancer, one must conclude that there is no rational at present to screen the general population, for a lack of appropriate tests.

## Introduction

The question of whether experience from pancreatic cancer surveillance programs can help us to detect pancreatic cancer in the general population does, obviously, imply that such screening will be able to identify patients at early enough stages to allow for curative surgery and, if necessary, adjuvant chemotherapy.

We therefore should first investigate the evidence from the screening programs in these risk populations for the detection of early cancer since here the risk to develop pancreatic cancer is higher. Consensus guidelines for individuals at risk (IAR) are developed by the international *Cancer of the Pancreas Screening* (CAPS) Consortium [1]. This group of IAR is better suited compared to other risk groups such as patients with chronic [2] or autoimmune [3] pancreatitis or patients with IPMN [4] that all are at risk to develop pancreatic cancer. The ground truth being surgical pathology demonstrating high-grade dysplasia or T1a invasive cancer as a reference. Screening programs usually consist of imaging at regular intervals, mostly by using MRI, with or without the addition of biomarkers such as CA 19–9 [5].

## Screening individuals at risk

**Fig. 1:**
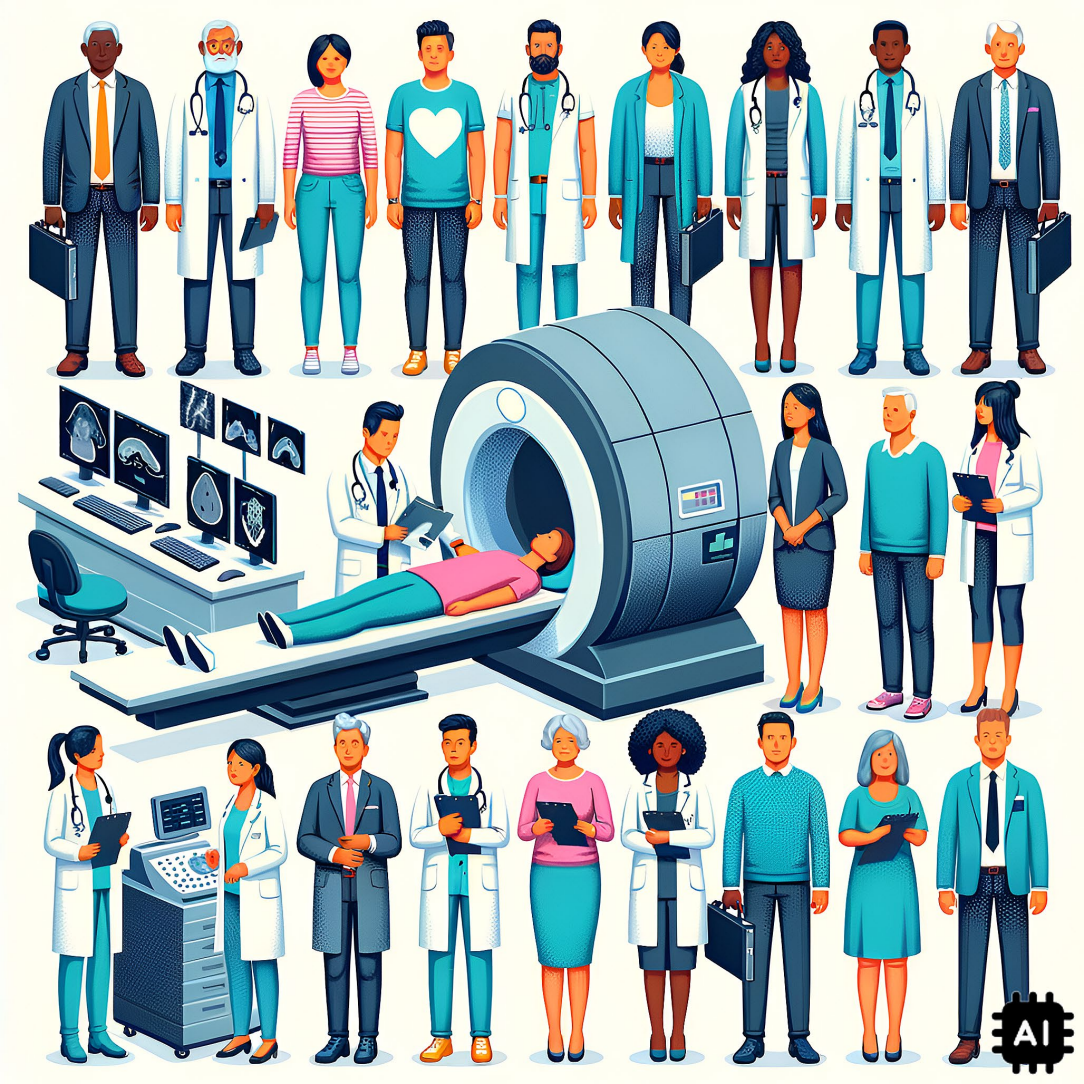
Screening for pancreatic cancer is a multidisciplinary approach

The aim of the screening of IAR is the detection and diagnosis of preneoplastic lesions, such as intraductal papillary mucinous neoplasia (IPMN), pancreatic intraepithelial neoplasia (PanIN) with high-grade dysplasia (HGD), or early-stage pancreatic ductal adenocarcinoma (PDAC). Several studies published by the CAPS Consortium have analyzed the prevalence of pancreatic abnormalities, preneoplastic lesions and pancreatic cancer, as well as the risk of malignant progression of IAR under surveillance [6, 7] Click or tap here to enter text. These studies show that IAR often have small cystic lesions [6] and that neoplastic progression (either HGD or pancreatic cancer) occurred in 7% with a progression rate of 1.6%/year [7]. Asymptomatic pancreatic cancers detected under surveillance were often identified in an early and resectable phase [8]. This led to a better 3- and 5-year survival in these IAR where a lesion was detected compared to symptomatic pancreatic cancer that developed outside the screening program [7, 9]. Other studies have however shown higher proportions of advance stage pancreatic cancer, including unresectable tumors, e.g. 11/28 in CAPS [9]. Surveillance thus seems to be effective in preventing death from pancreatic cancer [8], although some individuals may develop advanced cancer between the imaging intervals (depending on the risk typically between 6 and 12 months). Such cancers might perhaps be inherently more aggressive as has been shown for interval cancers for other types of tumors such as colorectal cancer [10] (Fig. 1).

## Health economy

There are few data on the health economic impact of the surveillance program in IAR. Cost-effectiveness analyses are used to inform decision makers on how to prioritize between different interventions to improve health. Assessing the cost-effectiveness of different surveillance programs will support policy making on how to optimally prioritize the allocation of health care resources to improve health for patients with pancreatic cancer.

Such analysis can be used to identify cost-effective surveillance program strategies, but also to identify risk populations (IAR), for which a specific surveillance program is cost-effective. As an example, it can be used to identify the lifetime pancreatic cancer risk threshold level, at which a specific surveillance strategy is cost-effective.

At current time, however, there is a significant knowledge gap on the health economic consequences of surveillance programs in IAR, and there is a need for new comprehensive cost-effectiveness studies, where all relevant consequences from a societal perspective are included, and where these are based on large population samples with long-term and complete follow-up [11]. Surveillance seems to be cost-effective [12], although the *number needed to treat* for preventing one PDAC is 135, thus rather high [13]. Therefore, the issue of overtreating has been raised meaning unnecessary surgery for finally a benign lesion [14, 15].

## Population-based studies

There are very few studies undertaken to screen an entire population for pancreatic cancer. Within the so-called SHIP study (Study of Health in Pomerania), 1’077 healthy individuals were subjected to whole-body MRI, and followed over a five-year period revealing no pancreatic cancer but cystic lesions (IPMN) in about 50% of those individuals [16].

Within the EPIC study, healthy individuals (405 cases and 416 matched controls) were followed up prospectively. Analyzing biological samples retrospectively, antibodies against bacteria from the oral cavity, especially *Porphyromonas gingivalis* could be linked to an increased risk for the development of PDAC [17]. This has not been used for screening the general population.

The Northern Sweden Health and Disease Study (NSHDS) is a prospective collection of blood samples, life-style and dietary factors from approximately 150,000 individuals, from a region in Northern Sweden. NSHDS contains pre-diagnostic blood samples from 275 future PDAC patients. Retrospective analysis of CA 19 − 9 in this cohort revealed that this marker became positive two years before PDAC diagnosis in some few patients, however, could only discriminate the future PDAC patients six month prior to overt diagnosis of the [18]. This underscores the critical role CA 19 − 9 plays as the only tumor marker for pancreatic cancer: even when using complex serum profiling, CA 19 − 9 had to be positive for determining a pancreatic cancer [19]. Moreover, data from the same cohort shows that fasting glucose levels start to increase already up to six years before the PDAC diagnosis in future patients and that this could be used as a means to find a high-risk population to enter into the surveillance programs in some settings [20]. At the same time data from this cohort also indicate that effective markers at the time of diagnosis could nevertheless be related to advance disease since these signatures disappear when moving further back in time from the diagnosis [21, 22]. From the PACYFIC register, it was concluded that the current CA19.9 cutoff was not predictive of HGD and pancreatic cancer, whereas a higher cutoff may decrease false-positive values. The role of CA19.9 monitoring should be critically appraised prior to implementation in surveillance programs and guidelines [23].

In a recent systematic review, clinical prediction models were developed to assess the risk of PDAC in the general population. The study evaluated a total of 15’848’100 individuals, of whom 58’313 developed PDAC (0.3%)^24^. The clinical risk factors included the usual suspects: cigarette smoking, chronic pancreatitis, and BMI, the CI running from 0.65 to 0.77 (diabetes mellitus), CI 0.75–0.89 (biomarkers and said risk factors) [24].

Newly onset diabetes mellitus (NOD) has been identified as a “red flag” for the development of PDAC [25]. While the scientific basis is sound [26, 27], the question arose as to whether it would be feasible to screen for pancreatic cancer in the general population all those who have NOD. A recent health economy analysis concluded that risk-based PAC screening in patients with NOD is likely to be cost-effective, at least in the United States if even a modest fraction (> 25%) of screen-detected patients with PAC are resectable [28]. Future studies should reassess the value of this intervention once clinical trial data become available.

As part of a metabolic syndrome, the fatty pancreas has been claimed to pave the road for pancreatic cancer as well. A recent systematic review of 2956 patients from 17 studies seem to confirm the potential association of a fatty pancreas and pancreatic cancer (6-fold higher probability of fatty pancreas among patients with pancreatic cancer). Limitations regarding the considerable statistical heterogeneity of the studies included leave this question open [29]. Furthermore, the authors themselves questioned the definition for the fatty pancreas since there are no guidelines or agreements amongst the radiologists [29].

Radiomics based on the primary lesion holds great potential for prognosis prediction. First-order entropy was significantly associated with the overall survival of PDAC and might improve the accuracy of current PDAC prognosis prediction [30].

## Conclusion

Taken together, whilst it appears to be possible to identify those amongst the individuals at risk who are on the path to develop pancreatic cancer, i.e. screening risk groups such as FPC/IAR and IPMN, the current knowledge does prevent us from extending such a surveillance – both via imaging and biomarkers - to the general population. Both due to lack of evidence, resources needed, and effect on possible outcome. This is in sharp contrast to screening programs for other cancers, such as mammography (breast cancer) and PSA screening (prostate cancer) or even screening colonoscopy (colorectal cancer) of the general public.

For the time being, the aim must be to thoroughly identify potential risk factors in patients rendering individuals then eligible for admission into surveillance such as familiar/hereditary pancreatic cancer, or IPMN. If a patient is diagnosed with chronic or autoimmune pancreatitis s/he ought to stay under regular observation, including new imaging with new symptoms (e.g. pain, weight loss) due to their underlying disease and the low risk of developing pancreatic cancer, however, these cannot count for the general (healthy) population.

The current line of research is aiming in two directions. First, identification of imaging features with help of artificial intelligence consistent of early pancreatic cancer [31]. Secondly, identifying the best liquid biopsy method indicating early pancreatic cancer during its inception [32, 33]. Both these tracks are followed at present through collaborative grants from the EU.

## Data Availability

No datasets were generated or analysed during the current study.
